# Advertising in Its Various Grades

**Published:** 1902-07-19

**Authors:** 


### ADVERTISING IN ITS VARIOUS GRADES.

THE ethical pronouncements of the *British Medical Journal* must always have a certain amount of interest, for it is only fair to believe that they are intended to express the sentiments of the Association of which that journal is the mouthpiece. It is, therefore, with considerable satisfaction that we find it urging, as we have urged again and again, that "it is very much to be desired that the General Medical Council, as well as the licensing bodies, should say distinctly what may not be done by medical practitioners in the matter of advertising."

The question has arisen in connection with the custom, which appears to exist in certain places, of advertising in the newspapers the names of the medical officers attached to the local "hydros" and similar institutions, and it is held that, in view of the determination of the General Medical Council to put a stop to the association of medical practitioners with medical aid societies which systematically practise canvassing and advertising for the purpose of securing patients, it would be "illogical to stop short at that point and not to prohibit advertising by medical practitioners themselves as well as the use of their names for advertising purposes by institutions, worked for profit, with which they may be connected." We cannot but agree with the sentiment, although we hesitate to accept the limitation implied by the words "worked for profit." It is the advertising "for the purpose of securing patients" which is the offence, the immediate profit made by the institutions by which the advertisements are ostensibly inserted being entirely beside the question; indeed, in one of the first cases dealt with by the Council it was admitted that not only was there no profit, but that a very heavy loss was suffered by the institution in question. But what about other, and quite as obvious, modes of advertising? Neither we nor the *British Medical Journal* can have any right, if we wish to be logical, to pillory certain gentlemen at Ilkley because their names appear in the advertisements of the hydropathic and other establishments with which they are connected, unless we are prepared to do the same in regard to many other forms of advertising, which have the effect of "securing patients," although they may not have been directly devised "for the purpose" of so doing. Are we so prepared? Those who excuse advertising would say that it is so evidently for the public convenience that, when a patient wishes to go to an institution conducted under the supervision of a reputable medical man, he should be able to find in an advertisement the information which he requires, that such advertising ought to be allowed. But, then, it may equally be said that it would be for the public convenience that a man wanting a specialist should be able to turn to the booksellers' lists in the daily and weekly papers and so find out who is "an authority" on the subject. Thus we soon find ourselves on the down grade, in the midst of writers of pamphlets written only that by being advertised they may advertise the writers, and to this position it is to be feared we have already nearly arrived.

Book-advertisement, even in the so-called medical papers, is too often mere self-advertisement, and it would certainly be doing little injustice, even to many men who stand pretty high in the profession, if we were to hint that they derive certain collateral advantages from the advertisement of their books quite apart from the increase of sales which may possibly be thereby induced—advantages not entirely disconnected with that "securing of patients" as to which the Medical Council has spoken so severely. Are we then prepared to stop all these advertisements? If, however, we are to persist in our effort to be logical, we must go still further, and must inquire whether the announcements of the names of the physicians and surgeons, the gynæcologists and ophthalmologists, the throat men, ear men, nose men, and skin men, which appear in the reports of our great hospitals and in the advertisements of their medical schools — pamphlets which are issued by tens of thousands every year—not to mention the publication of the names of the medical officers of certain special hospitals, are not also to be classed and condemned as advertisements.

Where are we to stop? At the one extreme we have the annual reports of the great hospitals, bristling with the names of baronets and great men —sorted out as physicians, surgeons, and specialists— reports carefully scanned by the wealthy when they desire a "second opinion"; then we come to the prospectus of the medical school, full of the names of specialists in every department, issued broadcast every summer among the middle classes and sometimes even inserted in the lay papers; then come the advertisements of sanatoria, and "open-air cures," of "hydros" and such-like institutions; and gradually we descend, by way of books and "treatments" and biscuits and dietetic substances—all with the names of the authors and inventors attached—until we reach things unmentionable. Who is to draw the line we know not, unless the Medical Council will intervene.

